# Poor Oral HIV Pre-Exposure Prophylaxis (PrEP) Persistence in an Integrated PrEP/STI Program in Malawi

**DOI:** 10.1007/s10461-025-04937-y

**Published:** 2025-11-29

**Authors:** Grace E. Mulholland, Mitch Matoga, Jane S. Chen, Esther Mathiya, Griffin J. Bell, Beatrice Ndalama, Tapiwa Munthali, Naomi Nyirenda, Naomi Bonongwe, Claire Pedersen, Edward Jere, Mina C. Hosseinipour, Zakaliah Mphande, Irving F. Hoffman, Sarah E. Rutstein

**Affiliations:** 1 Department of Epidemiology, University of North Carolina at Chapel Hill, Chapel Hill, NC USA; 2 UNC Project-Malawi, Lilongwe, Malawi; 3 Division of Infectious Diseases, Department of Medicine, University of North Carolina at Chapel Hill, Chapel Hill, NC USA; 4 Lighthouse Trust, Lilongwe, Malawi

**Keywords:** HIV prevention, PrEP, Sexually transmitted infections/diseases, Cohort studies, Africa, LMIC

## Abstract

The integration of HIV pre-exposure prophylaxis (PrEP) into STI services can improve PrEP uptake among a population at elevated risk of acquiring HIV. The effectiveness of PrEP relies on ongoing coverage during periods of HIV risk, however, and little is known about longitudinal PrEP use among people accessing PrEP through STI clinics in sub-Saharan Africa. In this study, we analyzed routine records data from people who newly initiated PrEP at an STI clinic in Lilongwe, Malawi in March-December 2022. We assessed PrEP persistence among clients who received Malawi’s standard-of-care PrEP services (*n* = 662) and reweighted the data to reflect the baseline distribution of age, sex, and PrEP indication among the full study population (*n* = 835). We used weighted generalized estimating equations to estimate the proportion of clients expected to persist on PrEP if all clients had received Malawi’s standard-of-care services. We also assessed predictors of persistence and described re-engagement in PrEP among clients who did not persist. We estimated that, had all clients received standard-of-care services, 17% (95% CI: 14%, 20%), 7% (95% CI: 6%, 10%), and 4% (95% CI: 3%, 5%) would have persisted on PrEP at 1, 3, and 6 months, respectively, and that 8% (95% CI: 5%, 11%) of those who did not persist on PrEP at 1 month would have re-engaged in PrEP services by 12 months. Persistence varied by age and PrEP indication. Our findings indicate very low PrEP persistence in this population and suggest opportunities to support ongoing PrEP use in settings with integrated PrEP/STI services.

**Supplementary Information:**

The online version contains supplementary material available at 10.1007/s10461-025-04937-y.

## Introduction

Substantial unmet need remains for effective HIV prevention strategies in sub-Saharan Africa, where approximately half of all new HIV infections globally occur [1]. Pre-exposure prophylaxis (PrEP) has shown considerable promise for HIV prevention [2], with daily oral PrEP reducing the risk of sexual HIV transmission by 90% or more [3, 4]. In recent years, HIV PrEP has been scaled up in several countries in sub-Saharan Africa. Still, HIV incidence remains high, with an estimated 650,000 people newly infected in the region in 2024 [1].

One strategy that may enhance the impact of PrEP is the integration of PrEP into sexual health services. People with sexually transmitted infections (STIs) are considered a priority population for PrEP, as HIV and other STIs share several behavioral risk factors [5–7], and STIs can increase biological susceptibility to HIV acquisition [8–10]. As such, PrEP/STI integration can help focus PrEP service delivery to people at elevated risk of HIV. Integration also presents opportunities to leverage existing clinical infrastructure for PrEP expansion and can add value and convenience for clients eligible for both PrEP and STI services [11]. In sub-Saharan Africa, many studies have described PrEP/STI integration within a third service setting (e.g., at a public health clinic) and the integration of STI services into PrEP-focused programs [12]. We are aware of only one study in the region, however, that describes the integration of PrEP into existing STI services [13].

It is important to understand patterns of longitudinal oral PrEP use in new implementation settings, including under the integration of PrEP into STI services. This is because the effectiveness of PrEP hinges on not just initiation of PrEP but on coverage throughout periods of ongoing HIV risk. Various measures of PrEP use have been described in sub-Saharan Africa, but many of these reports are from clinical trials or demonstration studies [14, 15]. In such settings, a narrow population focus, eligibility restrictions, financial incentives [14, 16], and participants’ interest in a study outcome [17] can influence PrEP use. It is therefore unclear how well the findings from such studies generalize to populations that access PrEP under standard clinical practices.

Routine health records data can be used to address this gap and gain insights into PrEP use among people receiving standard-of-care services [18–20]. Even if records are obtained from a setting where some clients are exposed to an intervention or research activities, we can estimate real-world PrEP use in the absence of these activities. This can be done by restricting longitudinal analyses to standard-of-care recipients and reweighting the data to account for intervention or study selection factors [21]. In the present study, we apply this approach to data from a PrEP program that was integrated into an STI clinic in Malawi.

Malawi has achieved a nearly 80% decline in HIV incidence since 2010, compared to a decline of roughly 55% in sub-Saharan Africa overall [22]. Following demonstration projects in 2019 and 2020, the Malawi Ministry of Health began providing PrEP in 2021 [23], and an estimated 54,000 people received PrEP in Malawi in 2024 [22]. Improvements in the implementation of HIV prevention strategies remain crucial, however, as 12,000 people were newly infected with HIV in Malawi in 2024 [22]. Understanding longitudinal PrEP use in populations with elevated HIV risk can help to inform high-yield strategies for HIV prevention. In addition, the development of approaches that use routine records data to produce insights for PrEP implementation can prove valuable not only in Malawi, but also in other places seeking to adopt or expand access to PrEP.

In this study, we leverage routine health records data to examine longitudinal PrEP use under standard-of-care services among people accessing PrEP at a public STI clinic in Lilongwe, Malawi. We estimate the probability that a client who receives Malawi’s standard-of-care PrEP services persists on PrEP at 1, 3, and 6 months. We also examine associations between baseline characteristics and PrEP persistence, and among clients who did not persist on PrEP at 1 month, we describe re-engagement in PrEP care in the 12 months following PrEP initiation.

## Methods

### Study Population

We defined the study population as all people who newly initiated oral PrEP at an STI clinic at Bwaila District Hospital in Lilongwe, Malawi between March 7 and December 31, 2022. In Malawi, people are eligible for PrEP if they are age 15 years or older, HIV seronegative, and at substantial risk of acquiring HIV. At the time of study conduct, groups considered to be at substantial risk were people with a current or recent STI, those with a partner who had an unsuppressed HIV viral load, people who bought or sold sex, and adolescent girls or young women ages 15–24 years with a partner 5 or more years older [24]. (More recent guidelines [25] have extended eligibility to include people who anticipate having an elevated risk of HIV acquisition in the next 3 months.)

While all clients in the study population initiated PrEP at the Bwaila STI clinic, the study population includes clients with various PrEP indications because PrEP service delivery was consolidated from multiple access points in the hospital. A few other observational studies took place at the STI clinic during the period of the present study; however, only one study (“ePrEP,” described below) specifically sought to engage PrEP users. We excluded, from our study population, clients who presented to the clinic due to the activities of that study (e.g., clients who sought services because they were referred by an ePrEP participant during ePrEP activities). All data for this study were abstracted from routine clinical records (i.e., PrEP client cards); as such, study eligibility required the presence of a PrEP client card at the clinic. Data were abstracted through July 14, 2023 to allow each client’s PrEP use to be assessed over a period of at least 6 months.

Among the 835 clients in the study population, 173 were enrolled in the enhanced PrEP STI Study (“ePrEP”). (ePrEP enrolled 174 clients; however, one lacked a linked PrEP client card and was therefore excluded from the present study.) ePrEP, described in detail elsewhere [13], examined the acceptability and feasibility of an enhanced integrated PrEP/STI service delivery model. To be eligible for ePrEP, clients had to be eligible for PrEP under Malawi’s contemporaneous guidelines and seeking STI services at the Bwaila STI clinic [24]. ePrEP participants received baseline and quarterly etiologic STI testing and assisted partner notification in addition to Malawi’s standard-of-care PrEP services. ePrEP participants were also asked to complete surveys and were compensated for study visits. The remaining 662 clients in the study population did not participate in ePrEP. These clients received only Malawi’s standard-of-care PrEP follow-up services: HIV testing, syndromic STI screening, PrEP refills as appropriate, and a review of their PrEP adherence.

PrEP follow-up visits were scheduled according to PrEP guidelines, at 30, 90, and 180 days (approximately 1, 3, and 6 months) after PrEP initiation. Subsequent visits were scheduled at 90-day intervals. The ePrEP staff abstracted PrEP client cards for all clients who accessed PrEP at the clinic during the study period, including ePrEP participants and clients who received only Malawi’s standard-of-care services.

The study was approved by the University of North Carolina at Chapel Hill Biomedical Institutional Review Board (21-2457) and Malawi National Health Services Research Committee (21/09/2777). Records data were abstracted with a waiver of informed consent.

### Coding PrEP Persistence

Our primary outcome was PrEP persistence. Though definitions vary, persistence typically incorporates the concepts of timely engagement in PrEP services and/or PrEP medication adherence [14, 26–30]. Both concepts are relevant to understanding ongoing PrEP coverage in the context of daily oral PrEP, as this modality requires periodic medication refills and regular pill-taking. At the time of this study, only daily oral PrEP was approved for use in Malawi [24].

Our primary definition of PrEP persistence was “missing fewer than 7 days of PrEP since a prior PrEP visit.” We used a consistent threshold for missed days of PrEP (7) regardless of the number of days in the follow-up visit interval because our intent was to capture the first instance when the client could have missed enough PrEP doses to substantially diminish the effectiveness of PrEP. The 7-day threshold was informed by evidence that protection against HIV does not cease immediately upon discontinuation of oral PrEP but wanes within 7 to 10 days [31, 32]. To explore the sensitivity of our estimates to this 7-day threshold, we also computed estimates with persistence defined as missing fewer than 14 or 21 days of PrEP since a prior visit.

We defined each client’s origin for follow-up as the date of PrEP initiation, and we assessed persistence over the following 180 days. Clients were coded as non-persistent on the first date on which they did not meet the definition of PrEP persistence, whether due to a lack of timely engagement in PrEP follow-up (based on visit dates) or low self-reported adherence (based on the number of PrEP doses reported as missed since the prior visit). Clients coded as non-persistent at any time point were considered non-persistent for the remainder of follow-up. For each client who was non-persistent at some point during follow-up, we used the number of days to non-persistence to determine the visit interval in which non-persistence occurred (i.e., between PrEP initiation and 1 month, between 1 month and 3 months, or between 3 and 6 months). Details of the persistence calculations are provided in Online Resource 1.

Our secondary outcome was re-engagement in PrEP within 12 months of PrEP initiation among clients who did not persist on PrEP at 1 month.

### Statistical Analysis

We summarized baseline characteristics of the study population overall and according to ePrEP participation. We used reweighted data from the 662 standard-of-care recipients to estimate PrEP persistence and PrEP re-engagement under standard-of-care services. We did not examine follow-up data for the ePrEP participants because the enhanced services and incentives provided by ePrEP may have influenced these clients’ PrEP use. The standard-of-care recipients were not, however, a simple random sample of all PrEP initiators at the clinic. This is because ePrEP only recruited people who presented with STI symptoms. As a result, people with STI symptoms were underrepresented among standard-of-care recipients. To account for this and produce estimates generalizable to all PrEP initiators at the clinic, we computed inverse probability weights [33] to balance baseline differences in age, sex, and PrEP indication between ePrEP participants and standard-of-care recipients. The reweighted data from standard-of-care recipients depict the expected pattern of PrEP use at the clinic under the hypothetical scenario where all PrEP initiators had received standard-of-care PrEP services. Additional details of the weighting procedure are provided in Online Resource 2, along with a table that shows the distribution of client characteristics at PrEP enrollment with the inverse probability weights applied.

We estimated the marginal probability of our primary outcome, PrEP persistence, at 1, 3, and 6 months. We also estimated the probability of PrEP persistence at 1, 3, and 6 months within strata of baseline covariates, and we computed probability ratios (PRs) to estimate associations between baseline characteristics and PrEP persistence. We performed these analyses using generalized estimating equations (GEE), which allowed us to account for within-client correlation across repeated assessments of persistence (i.e., at 1, 3, and 6 months). In fitting the GEE models, we assumed an exchangeable correlation structure, specified a log-binomial outcome distribution, and applied the client-level inverse probability weights previously described. We used robust standard errors to compute all 95% confidence intervals.

For our secondary outcome, re-engagement in PrEP after non-persistence among those who did not persist on PrEP at 1 month, we recomputed the inverse probability weights among the subset of clients who did not persist at 1 month and who initiated PrEP early enough in the study period that any re-engagement in PrEP services within 12 months could have been observed. We used GEE with the recomputed weights applied to estimate the probability of PrEP re-engagement among this subset of clients.

Analyses were conducted in R version 4.3.0.

## Results

Just under half (45.1%) of the 835 clients were female (Table 1), and the median age was 28 (interquartile range [IQR]: 24, 36). The most common PrEP indication, recorded for 62.4% of clients, was a current or recent STI. Nearly half of clients (46.7%) had a partner with an unsuppressed HIV viral load, 30.7% bought or sold sex, and 4.9% were adolescent girls or young women with a partner who was older by 5 or more years. Though clients could report multiple PrEP indications, the median number was 1 (IQR: 1, 2). The most commonly co-occurring indications were current or recent STI and buying or selling sex (*n* = 216; 25.9% of clients) and current or recent STI and partner with an unsuppressed HIV viral load (*n* = 107; 12.8% of clients). Other combinations of co-occurring indications were reported by less than 3% of clients.

With respect to the characteristics assessed, standard-of-care recipients and ePrEP participants were generally similar, apart from the distribution of baseline PrEP indications. Compared to ePrEP participants, standard-of-care recipients were less likely to have had a current or recent STI (53.5% vs. 96.5%), less likely to buy or sell sex (24.5% vs. 54.3%), and more likely to have a partner with an unsuppressed HIV viral load (54.8% vs. 15.6%). Adolescent girls and young women with an older partner comprised 3.8% of the standard-of-care recipients and 9.2% of ePrEP participants.

**Table 1:** Client characteristics at the time of PrEP initiation

	All new PrEP initiators (*N* = 835)		Participated in ePrEP?			
			No; received standard-of-care services (*N* = 662)		Yes; received enhanced services^a^ (*N* = 173)	
	*n*	%	*n*	%	*n*	%
*Female*	377	45.1	310	46.8	67	38.7
*Age*						
15–24 years	243	29.1	177	26.7	66	38.2
25–34 years	345	41.3	266	40.2	79	45.7
35–44 years	179	21.4	153	23.1	26	15.0
45 + years	68	8.1	66	10.0	2	1.2
*Among female clients: Pregnancy and breastfeeding status* ^b^						
Pregnant	16	4.3	16	5.3	0	0.0
Breastfeeding	37	10.0	34	11.2	3	4.5
Not pregnant or breastfeeding	317	85.7	254	83.6	63	95.5
Missing (not indicated on form)	7		6		1	
*Among male clients: Circumcision status* ^c^						
Circumcised	193	42.9	151	43.8	42	40.0
Missing (not indicated on form)	8		7		1	
*Current STI or STI in the past 6 months*	521	62.4	354	53.5	167	96.5
*Partner with unsuppressed HIV viral load*	390	46.7	363	54.8	27	15.6
*Buys or sells sex*	256	30.7	162	24.5	94	54.3
*Among adolescent girls and young women (age 15–24): Partner 5 + years older* ^d^	41	33.9	25	28.7	16	47.1
*Number of PrEP indications* ^e^						
0	38	4.6	37	5.6	1	0.6
1	414	49.6	362	54.7	52	30.1
2	355	42.5	247	37.3	108	62.4
3	28	3.4	16	2.4	12	6.9
4	0	0.0	0	0.0	0	0.0
*Received PrEP readiness education*	819	98.1	650	98.2	169	97.7
*Acute HIV infection assessment*						
Acute infection not suspected	188	23.2	145	22.7	43	24.9
No assessment done	624	76.8	494	77.3	130	75.1
Missing (not indicated on form)	23		23			
*Kidney risk assessment* ^f^						
Screened; determination of high risk	0	0.0	0	0.0	0	0.0
Screened; determination of low risk	109	13.4	92	14.4	17	9.8
Not screened	702	86.6	546	85.6	156	90.2
Missing (not indicated on form)	24		24		0	
*Hepatitis B testing*						
Reactive result	1	0.1	1	0.2	0	0.0
Non-reactive result	833	99.8	661	99.8	172	99.4
No test administered	1	0.1	0	0.0	1	0.6

*PrEP* Pre-exposure prophylaxis, *STI* Sexually transmitted infection

^a^ePrEP participants received enhanced STI services (i.e., baseline and quarterly etiologic STI testing and assisted partner notification) in addition to the standard-of-care services. They also completed bio-behavioral surveys and received financial incentives

^b^Denominator for percentages: female clients

^c^Denominator for percentages: male clients

^d^Denominator for percentages: adolescent girls and young women (female clients ages 15–24)

^e^Among: Buys or sells sex, current STI or STI in the past 6 months, partner with unsuppressed HIV viral load, or adolescent girl or young woman with partner 5 + years older

^f^Malawi PrEP guidelines call for baseline creatine clearance testing prior to PrEP initiation for clients age > 50, hypertension, diabetes mellitus, body mass index < 18.5, other nephrotoxic medication, or any signs or symptoms suggestive of renal impairment

Data are from routine PrEP client cards for 835 clients who newly initiated PrEP at an STI clinic in Lilongwe, Malawi in March-December 2022

**Fig. 1:**
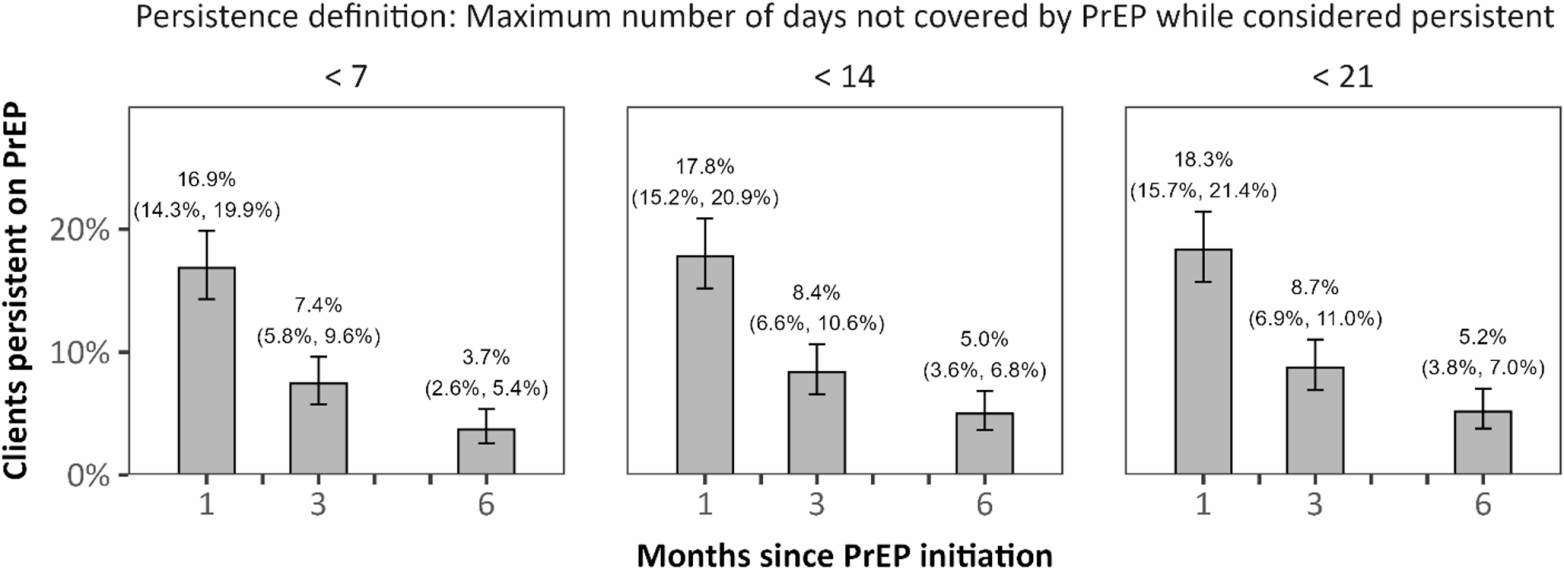
PrEP persistence under 3 persistence definitions. Bars show the percentage of clients estimated to persist on PrEP under Malawi’s standard-of-care PrEP services when persistence is defined as less than 7, 14, or 21 days not covered by PrEP since a prior PrEP visit. Data were reweighted to reflect the baseline distribution of age, sex, and PrEP indication among all 835 PrEP initiators, and robust standard errors were used in computing 95% confidence intervals (presented parenthetically)

Among the 662 standard-of-care recipients, 123 (19.0%) persisted on PrEP at 1 month, 56 (8.5%) persisted at 3 months, and 28 (4.2%) persisted at 6 months. After reweighting data from these clients to represent the full study population, we estimated that, had all clients received standard-of-care PrEP services, 16.9% (95% CI: 14.3%, 19.9%), 7.4% (95% CI: 5.8%, 9.6%), and 3.7% (95% CI: 2.6%, 5.4%) would have persisted on PrEP at 1, 3, and 6 months, respectively (Fig. 1). These results were computed under our primary definition of PrEP persistence: missing fewer than 7 days of PrEP since a prior PrEP visit. Estimates were similar under the alternative persistence thresholds we considered (i.e., missing fewer than 14 or 21 days of PrEP since a prior PrEP visit).

Female clients were 1.34 (95% CI: 0.96, 1.86) times as likely as male clients to persist on PrEP at 1 month, though the association between sex and PrEP persistence waned over time (Table 2). Older age was associated with a higher probability of PrEP persistence at each time point; for example, at 1 month, we estimated a 36.0% (95% CI: 26.1%, 49.5%) probability of persistence among clients 45 years of age or older, which was 2.63 (95% CI: 1.71, 4.06) times the probability of persistence among clients ages 25 to 34. Compared to female clients who were not pregnant or breastfeeding, pregnant clients were 0.61 (95% CI: 0.16, 2.31) times as likely to persist on PrEP at 1 month.

**Table 2:** PrEP persistence by baselinecharacteristics. Persistence was estimated under Malawi’s standard-of-care PrEPservices

	Percentage of clients persistent^a^ (95% CI^b^) at:			Persistence probability ratio (95% CI) at:			
	1 month	3 months	6 months	1 month		3 months	6 months
*Overall*	16.9 (14.3, 19.9)	7.4 (5.8, 9.6)	3.7 (2.6, 5.4)				
*Sex*							
Female	19.6 (15.7, 24.4)	7.5 (5.1, 10.9)	3.7 (2.2, 6.3)	1.34 (0.96, 1.86)		1.01 (0.60, 1.69)	0.99 (0.48, 2.08)
Male	14.6 (11.5, 18.7)	7.4 (5.2, 10.5)	3.7 (2.2, 6.2)	1.00		1.00	1.00
*Age*							
15–24 years	13.8 (9.6, 19.7)	5.4 (3.0, 9.6)	3.3 (1.6, 7.0)	1.01 (0.64, 1.60)		0.77 (0.37, 1.58)	1.14 (0.42, 3.05)
25–34 years	13.7 (10.2, 18.3)	7.0 (4.6, 10.6)	2.9 (1.5, 5.6)	1.00		1.00	1.00
35–44 years	19.4 (14.2, 26.7)	7.4 (4.2, 12.7)	4.3 (2.1, 9.0)	1.42 (0.93, 2.19)		1.05 (0.53, 2.10)	1.49 (0.56, 3.95)
45 + years	36.0 (26.1, 49.5)	17.0 (10.1, 28.6)	7.1 (3.0, 16.6)	2.63 (1.71, 4.06)		2.43 (1.25, 4.75)	2.44 (0.84, 7.10)
*Among female clients: Pregnancy and breastfeeding status* ^c^							
Pregnant	11.9 (3.2, 43.9)	0.0	0.0	0.61 (0.16, 2.31)		–	–
Breastfeeding	24.9 (13.9, 44.6)	5.6 (1.5, 21.8)	0.0	1.28 (0.68, 2.41)		0.68 (0.17, 2.77)	–
Not pregnant or breastfeeding	19.4 (15.3, 24.8)	8.3 (5.6, 12.3)	4.4 (2.9, 6.8)	1.00		1.00	–
*Among male clients: Circumcision status*							
Circumcised	11.6 (7.7, 17.5)	7.3 (4.3, 12.5)	4.0 (2.3, 7.2)	0.71 (0.42, 1.18)		0.95 (0.47, 1.92)	1.12 (0.41, 3.06)
Not circumcised	16.4 (12.0, 22.4)	7.7 (4.9, 12.3)	3.6 (2.1, 6.1)	1.00		1.00	1.00
*STI at PrEP initiation visit or in the prior 6 months*							
Yes	10.4 (8.5, 12.7)	4.7 (3.5, 6.4)	2.4 (1.6, 3.7)	0.38 (0.27, 0.53)		0.39 (0.23, 0.67)	0.41 (0.19, 0.89)
No	27.6 (23.1, 33.0)	12.0 (8.9, 16.1)	5.8 (3.8, 9.1)	1.00		1.00	1.00
*Partner with unsuppressed HIV viral load*							
Yes	27.0 (23.1, 31.6)	12.6 (9.8, 16.2)	6.6 (4.6, 9.5)	3.35 (2.23, 5.05)		4.21 (2.14, 8.28)	5.71 (1.96, 16.64)
No	8.0 (6.3, 10.3)	3.0 (2.0, 4.5)	1.2 (0.6, 2.3)	1.00		1.00	1.00
*Buys or sells sex*							
Yes	5.4 (3.6, 8.0)	1.5 (0.7, 3.1)	1.1 (0.4, 2.6)	0.24 (0.13, 0.46)		0.15 (0.05, 0.49)	0.22 (0.05, 0.93)
No	22.0 (19.1, 25.4)	10.1 (8.1, 12.6)	4.9 (3.5, 6.8)	1.00		1.00	1.00
*Among adolescent girls or young women (ages 15–24): Partner 5 + years older*							
Yes	12.4 (6.6, 23.2)	2.5 (0.7, 8.8)	2.5 (0.7, 8.8)	0.91 (0.29, 2.88)		0.43 (0.05, 3.81)	2.02 (0.13, 32.07)
No	13.6 (8.5, 21.7)	5.8 (2.7, 12.5)	1.2 (0.3, 5.8)	1.00		1.00	1.00

*CI* Confidence interval, *PrEP* Pre-exposure prophylaxis, *STI* Sexually transmitted infection

^a^Persistence was defined as missing fewer than 7 days of PrEP since a prior PrEP visit

^b^Data were reweighted to reflect the baseline distribution of age, sex, and PrEP indication among all 835 PrEP initiators, and robust standard errors were used in computing 95% confidence intervals (presented parenthetically)

^c^Dashes (–) indicate comparisons where, within one or both levels of the characteristic, 0 clients persisted on PrEP

Data are from routine PrEP client cards for 835 clients who newly initiated PrEP at an STI clinic in Lilongwe, Malawi in March-December 2022

**Fig. 2:**
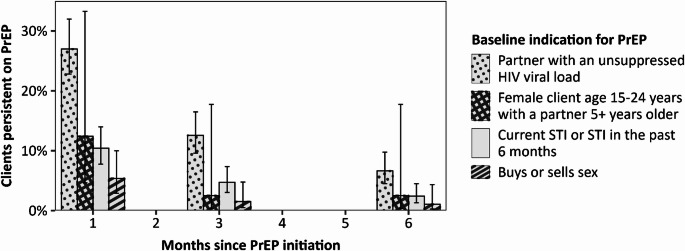
Persistence by baseline PrEP indication. Bars show the percentage of clients with each indication for PrEP estimated to persist on PrEP under Malawi’s standard-of-care PrEP services. Clients with multiple indications are included in each applicable bar. Data were reweighted to reflect the baseline distribution of age, sex, and PrEP indication among all 835 PrEP initiators, and robust standard errors were used in computing 95% confidence intervals (indicated by error bars)

PrEP persistence also differed by baseline PrEP indication (Fig. 2). Clients with a current or recent STI were less likely than others to persist on PrEP at 1 month (PR = 0.38; 95% CI: 0.27, 0.53). Associations were similar at 3 and 6 months. Buying or selling sex was also associated with lower persistence; the probability of persistence among those who bought or sold sex was 0.24 (95% CI: 0.13, 0.46), 0.15 (95% CI: 0.05, 0.49), and 0.22 (95% CI: 0.05, 0.93) times the probability of persistence among other clients at 1, 3, and 6 months, respectively. Clients who had a partner with an unsuppressed HIV viral load were much more likely than others to persist on PrEP at each visit interval. At 1, 3, and 6 months, respectively, clients with a baseline PrEP indication of a partner with unsuppressed HIV were 3.35 (95% CI: 2.23, 5.05), 4.21 (95% CI: 2.14, 8.28), and 5.71 (95% CI: 1.96, 16.64) times as likely as other clients to have persisted on PrEP. Among adolescent girls and young women, the association between having an older partner and persisting on PrEP varied over time, though all confidence intervals were wide and contained the null value.

We also computed sex-stratified probabilities of persistence and PRs by PrEP indication (Online Resource 3). Some stratified PRs could not be estimated due to sparse cells; otherwise, most PRs estimating the association between PrEP indication and persistence were similar between male and female clients. One exception was at 1 month, where the negative association between buying or selling sex and PrEP persistence was more pronounced among female clients (PR = 0.08; 95% CI: 0.02, 0.39) than among male clients (PR = 0.31; 95% CI: 0.18, 0.53). Also, at 3 months, the positive association between having a partner with an unsuppressed HIV viral load and PrEP persistence was stronger among female clients (PR = 7.03; 95% CI: 2.12, 23.34) than among male clients (PR = 3.77; 95% CI: 2.05, 6.95).

About 40% (*n* = 354; 42.4%) of clients initiated PrEP early enough in the study period that their engagement in PrEP services could be assessed for at least 12 months. This included 270 standard-of-care recipients. Among these clients, 37 (13.7%) persisted on PrEP at 1 month, while 233 (86.3%) did not. Among the 233 who did not persist at 1 month, 24 (10.3%) returned within 12 months of initiating PrEP. After reweighting to account for baseline differences between standard-of-care recipients and ePrEP participants, we estimated that, under standard-of-care PrEP services, those who did not persist on PrEP at 1 month had a 7.8% (95% CI: 5.2%, 11.5%) probability of re-engaging in PrEP services within 12 months of initiating PrEP.

## Discussion

Progress towards reducing HIV incidence in Malawi has slowed and fallen short of targets in recent years [24]. Though PrEP is being rapidly scaled up, our findings suggest that under Malawi’s standard-of-care PrEP follow-up services, the benefits of PrEP will not be fully realized for a large proportion of people initiating PrEP. Though some proportion of PrEP initiators may not remain at elevated risk of acquiring HIV for an extended period, even in the short term, we estimated that less than 20% persisted on PrEP at one month, and less than 5% persisted at 6 months.

Though comparisons of persistence estimates are complicated by differences in how persistence has been defined across studies, the probability of PrEP persistence at this STI clinic appears to be substantially lower than in other populations in sub-Saharan Africa where longitudinal oral PrEP use has been described [27, 29, 34–46]. This finding is consistent with a meta-analysis [14] which found that PrEP discontinuation and sub-optimal adherence were more common in real-world implementation settings than in clinical trials and demonstration projects. Furthermore, the frequency of PrEP re-initiation/re-engagement has varied across populations in sub-Saharan Africa [39, 45, 46]. At this STI clinic, we found that re-engagement in PrEP services was uncommon among non-persistent PrEP users. While silent transfers (i.e., unrecorded PrEP engagement at other PrEP distribution sites) may have biased our persistence or re-engagement estimates downwards, we expect that such transfers were uncommon, given the limited number of facilities providing PrEP during the study period and the small number of transfers recorded in the STI clinic’s register. Due to the dearth of evidence concerning longitudinal PrEP use in STI clinics in sub-Saharan Africa [12], it is unclear whether the patterns of PrEP persistence and re-engagement estimated here are comparable to what would be observed at other STI clinics in the region.

Our findings of poor PrEP persistence and infrequent re-engagement may be related to the implementation setting, where PrEP was integrated into STI services. We found that a current or recent STI was associated with a lower probability of persistence, and more than half of clients at this clinic had a current or recent STI when initiating PrEP. Though we could not examine drivers of this association, one possibility is that the integration of PrEP into STI services encourages PrEP use when services conveniently co-occur, but once STI symptoms resolve, motivation for continuing PrEP wanes. It is also possible that instructions regarding the intended durations of medication use are conflated under the simultaneous provision of PrEP and STI treatment. Integrated PrEP programs could address these challenges by improving the convenience of PrEP services, ensuring clear instructions, and highlighting the value of PrEP even in the absence of STI symptoms.

The second-most common indication for PrEP was having a partner with an unsuppressed HIV viral load. Of the four indications examined, this was the only one consistently associated with a higher probability of PrEP persistence. Elsewhere, perception of greater HIV risk has been associated with higher PrEP adherence [47]. If clients with a partner with an unsuppressed HIV viral load perceive themselves to be at a consistently elevated risk of HIV acquisition, this may explain why they were more likely to persist on PrEP. We did, however, expect persistence to decline somewhat among these clients at around 6 months. This is because according to Malawi’s contemporaneous PrEP guidelines, unless a client had additional indication(s) for PrEP, PrEP would have no longer been indicated once the client’s partner was on stable, effective HIV treatment [24]. While persistence did decline in this group, we did not have data that would allow us to identify what proportion of non-persistence was attributable to a client’s loss of PrEP indication due to the partner’s viral suppression.

We acknowledge that persistent PrEP use does not necessarily equate to “prevention-effective” PrEP use [48]. We were unable to identify what proportion of non-persistence was attributable to loss of PrEP indication or other fluctuations in HIV risk. In Malawi, clients are advised to inform their provider when they wish to discontinue PrEP [24], but across this study population, no losses of indication were recorded on the PrEP client cards. We presume that some proportion of clients who disengaged from PrEP services had appropriately discontinued PrEP in accordance with a loss of indication but did not return to the STI clinic to allow their loss of indication to be recorded. The imperfect correlation between PrEP persistence and prevention-effective PrEP use does not, however, negate the utility of persistence estimates for making programmatic recommendations to support ongoing PrEP use. The persistence estimates seem particularly useful in this population, given what appears to be infrequent re-engagement with any PrEP services.

Our analyses were limited by the information recorded in the PrEP client cards. This required us to make assumptions when coding PrEP persistence. We assumed, for example, that PrEP doses recorded as “missed” remained available to a client. In any cases where missed doses were not available, we would have overestimated a client’s PrEP supply and may have, as a result, misclassified a non-persistent PrEP user as persistent. We examined the potential impact of this assumption in sensitivity analyses and found, however, that including missed doses in the PrEP supply calculation had a negligible effect on the estimates (see Online Resource 4). Furthermore, though the effectiveness of PrEP may vary according to differences in the timing of missed PrEP doses (e.g., 7 sequential doses vs. 1 dose per week for 7 weeks) [49], we were unable to account for these differences in our persistence definition, as the timing of missed doses was not captured in the client cards. We also did not have information on additional factors that may be relevant to PrEP persistence, such as socioeconomic status, educational attainment, time-varying HIV risk behaviors, and HIV risk perception. Despite these limitations, our study demonstrates a useful approach for describing and comparing longitudinal PrEP use in a real-world implementation setting under common constraints of routine records data. Approaches such as this that leverage routine records data for surveillance and research purposes may prove particularly important in light of recent disruptions to funding for PrEP [50] and other aspects of the global HIV response [51].

High rates of acute HIV infection have been reported among people seeking STI services in Malawi [52], underscoring the importance of HIV prevention strategies such as PrEP in this population. HIV risk behaviors can be unpredictable [53, 54], and among populations at elevated HIV risk, including those presenting for STI services, persistent PrEP use confers protection in the event of unanticipated HIV exposure. While we do not know the HIV status of people who disengaged from PrEP services, no HIV seroconversions were recorded during follow-up among clients in this study who remained engaged in PrEP.

## Conclusions

The effectiveness of daily oral PrEP relies on continued engagement in PrEP services and PrEP adherence among people at ongoing risk of HIV. Understanding patterns of PrEP use in routine clinical settings can inform optimized strategies for PrEP implementation and calibrate expectations for the impact of PrEP on HIV incidence. In an urban STI clinic offering daily oral PrEP in Malawi, we estimated that under standard-of-care PrEP services, only about 1 in 6 PrEP initiators persist on PrEP at 1 month, and 1 in 27 persist at 6 months. Persistence estimates were sensitive to age and reason for initiating PrEP; therefore, expectations and interventions to support longitudinal PrEP use should consider demographics and the distribution of HIV risk factors in the client population. While the integration of PrEP into STI services may be an efficient strategy for improving uptake of PrEP among people at elevated risk of HIV, additional strategies are needed to achieve sustained preventative benefits.

## Supplementary Information

Below is the link to the electronic supplementary material.

Supplementary Material 1

Supplementary Material 2

Supplementary Material 3

Supplementary Material 4

## Data Availability

The data and code relevant to the findings of this study are available from the corresponding author upon reasonable request.
