# Supplementary material for: Poor Oral HIV Pre-Exposure Prophylaxis (PrEP) Persistence in an Integrated PrEP/STI Program in Malawi
Source: AIDS Behav. 2025 Nov 29;30(5):1327–37. doi: 10.1007/s10461-025-04937-y (PMC13167809; doi:10.1007/s10461-025-04937-y)
Supplement: Supplementary file 1 — Supplementary Material 1 [file 10461_2025_4937_MOESM1_ESM.pdf]

Mulholland GE, Matoga M, Chen JS, Mathiya E, Bell GJ, Ndalama B, Munthali T, Nyirenda N, Bonongwe N, Pedersen C, Jere E, Hosseinipour MC, Mphande Z, Hoffman IF, Rutstein SE. Poor oral HIV pre-exposure prophylaxis persistence in an integrated PrEP/STI program in Malawi. AIDS and Behavior.

Corresponding author: Grace E. Mulholland (gem@unc.edu); Department of Epidemiology, University of North Carolina at Chapel Hill, Chapel Hill, North Carolina, United States

Online Resource 1. Details of persistence outcome coding

Under our primary outcome definition, a lack of timely engagement was determined if a PrEP follow-up visit occurred 7 or more days after a client had, in expectation, depleted their PrEP supply. For each visit, we determined the client's expected date of PrEP supply depletion by adding the number of PrEP tablets dispensed to the number of tablets from their prior visit that they reported as "missed." This approach assumed that any PrEP tablets reported as "missed" remained available to the client. We also assessed adherence at each visit, coding poor adherence if a client reported missing 7 or more doses of PrEP since their previous visit. We assumed that all PrEP doses not reported as "missed" were taken and that they were taken daily, consistent with prescription instructions.

To compute persistence at each follow-up visit, we first coded a binary indicator variable for timely engagement in PrEP follow-up. To code the timely engagement indicator variable (e), we subtracted the PrEP supply a client had upon departure from their previous PrEP visit from the number of days (d) that had elapsed by the subsequent PrEP visit.

We assumed that at the end of each PrEP visit, a client departed with an available PrEP supply equal to the number of PrEP tablets dispensed at that visit (g) plus the number of PrEP doses (tablets) that they reported having missed since their prior visit (m). As no missed doses could be reported at the PrEP initiation visit (i.e., $m_{t=1} = 0$), the available PrEP supply upon departing the PrEP initiation visit was simply the number of PrEP tablets dispensed (g).

With t indexing PrEP visits (1, 2, 3…), the following equation represents how e was computed at each PrEP follow-up visit, under a persistence definition of < 7 days not covered by PrEP since the prior visit:

$$e_t = \begin{cases} 1 & \text{if } d_t - d_{t-1} - (g_{t-1} + m_{t-1}) < 7 \\ 0 & \text{otherwise} \end{cases}$$

where:

$e_t = 1$ indicates timely engagement at visit t, and

$e_t = 0$ indicates late engagement at visit t or disengagement.

We next coded a medication adherence indicator variable (a) based on the number of PrEP doses missed since the prior PrEP visit, as reported at the current follow-up PrEP visit. Under a persistence definition of < 7 days not covered by PrEP since the prior visit, this is represented by:

$$a_t = \begin{cases} 1 & \text{if } m_t < 7 \\ 0 & \text{otherwise} \end{cases}$$

where:

$a_t = 1$ indicates sufficient adherence reported at visit t, and

$a_t = 0$ indicates insufficient adherence reported at visit t.

For each client, at every PrEP follow-up visit, we computed a binary outcome for persistent PrEP use (p) as the product of the indicator variables representing timely engagement in PrEP follow-up and medication adherence:

$$p_t = e_t a_t$$

where:

$p_t = 1$ indicates persistence at visit t, and

$p_t = 0$ indicates persistence at visit t.

With the persistence variables computed, we coded a continuous variable for the number of days with documented persistence. To do this, we identified the client's latest visit at which the current and all previous values of p_t indicated persistent PrEP use, and we computed the number of days between PrEP initiation and this latest visit where PrEP persistence was documented.

We used this continuous variable to assess persistence at 30, 90, and 180 days since PrEP initiation. We allowed a grace period of 5 days to accommodate scheduling variations that could result in a PrEP visit occurring before the exact interval prescribed under Malawi's standard PrEP follow-up schedule. Specifically, we considered clients to be persistent at the assessment time (30, 90, or 180 days) if they satisfied the persistence definition at the assessment time or in the 5 days preceding the assessment time.

<u>Example</u>

Consider the following scenario:

- A client received 30 PrEP tablets at their PrEP initiation visit ($t = 1$).
- The client returned to the clinic for their second PrEP visit ($t = 2$) 36 days after visit 1. At this visit:
    - they reported having missed 2 PrEP doses since their prior visit ($t = 1$).
    - they received 60 PrEP tablets.
- The client returned to the clinic for their second PrEP visit ($t = 3$) 56 days after visit 2.
    - At this visit, they reported having missed 7 PrEP doses since their prior visit ($t = 1$). In this scenario:

For this scenario:

$e_{t=2} = 1,$ as $36 - (30 - 0) < 7$, indicating timely engagement in PrEP follow-up at this visit.

$a_{t=2} = 1,$ as $2 < 7$, indicating sufficient adherence.

$p_{t=2} = 1 \times 1 = 1$, indicating persistence at visit 2.

$e_{t=3} = 1,$ as $56 - (60 + 2) < 7$, indicating timely engagement in PrEP follow-up at this visit.

$a_{t=3} = 0,$ as $7 = 7$, indicating insufficient adherence.

$p_{t=3} = 1 \times 0 = 1$, indicating non-persistence at visit 3.

Days with documented persistence = 36; the client would be considered persistent at 30 days and non-persistent at later time points.
