# Supplementary material for: Poor Oral HIV Pre-Exposure Prophylaxis (PrEP) Persistence in an Integrated PrEP/STI Program in Malawi
Source: AIDS Behav. 2025 Nov 29;30(5):1327–37. doi: 10.1007/s10461-025-04937-y (PMC13167809; doi:10.1007/s10461-025-04937-y)
Supplement: Supplementary file 2 — Supplementary Material 2 [file 10461_2025_4937_MOESM2_ESM.pdf]

Mulholland GE, Matoga M, Chen JS, Mathiya E, Bell GJ, Ndalama B, Munthali T, Nyirenda N, Bonongwe N, Pedersen C, Jere E, Hosseinipour MC, Mphande Z, Hoffman IF, Rutstein SE. Poor oral HIV pre-exposure prophylaxis persistence in an integrated PrEP/STI program in Malawi. AIDS and Behavior.

Corresponding author: Grace E. Mulholland (gem@unc.edu); Department of Epidemiology, University of North Carolina at Chapel Hill, Chapel Hill, North Carolina, United States

Online Resource 2. Computation of inverse probability weights

For each recipient of Malawi's standard-of-care PrEP services, we computed the inverse probability weight as their marginal probability of receiving standard-of-care PrEP services divided by their probability of receiving standard-of-care PrEP services conditional on informative baseline covariates. We estimated probabilities using logistic regression.

For all standard-of-care recipients, the marginal probability of receiving standard-of-care PrEP services was:

$$P(A=1)=\frac{e^{\beta_0}}{1+e^{\beta_0}}=0.793$$

where $A=1$ indicates receipt of the standard of care vs. participation in ePrEP ($A=0$).

The conditional probability of receiving standard-of-care PrEP services was modeled as a function of the client's age, sex, and indication for PrEP, i.e.:

$$P(A=1|b,c,d,g,h,j)=\frac{e^{\beta_0+\beta_1 b+\beta_2 c+\beta_3 d+\beta_4 g+\beta_4 h+\beta_4 j}}{1+e^{\beta_0+\beta_1 b+\beta_2 c+\beta_3 d+\beta_4 g+\beta_4 h+\beta_4 j}}$$

where:

- b = age (continuous)
- c = sex (binary)
- d= at PrEP initiation, having an STI at baseline or in the prior 6 months (binary)
- g = at PrEP initiation, having a partner with an unsuppressed HIV viral load (binary)
- h = at PrEP initiation, being an adolescent girl or young woman with a partner at least 5 years older (binary)

- j = at PrEP initiation, reporting buying or selling of sex (binary)

The stabilized weight (w) for each standard-of-care recipient (i) was computed as:

$$w_i = \frac{P(A = 1)}{P(A = 1 | b_i, c_i, d_i, g_i, h_i, j_i)}$$

The mean of the stabilized weights was 1.00 (min = 0.80, max = 2.06).

Assuming correct model specification, the reweighted data from the standard-of-care recipients represent patterns of longitudinal PrEP use that we would have expected to observe had all 835 clients in the study population received Malawi's standard-of-care PrEP services.

The Table below shows the unweighted distribution of client characteristics (as provided in the main text) alongside the distribution of client characteristics with weights applied. For ease of comparison to the full (unweighted) population of PrEP initiators, we applied unstabilized weights* to show the expected Ns among all PrEP initiators. (Note that the weighted percentages are the same, whether unstabilized or stabilized weights are used). The table confirms the expected similarity in the distributions of age, sex, and baseline PrEP indication between the unweighted and weighted data.

Table. Client characteristics at the time of PrEP initiation: unweighted and with weights applied.

	All new PrEP initiators, unweighted		All new PrEP initiators, weights applied	
	n	%	n	%
Female	377	45.1	378	45.2
Age				
15 - 24 years	243	29.1	255	30.5
25 - 34 years	345	41.3	334	39.9
35 - 44 years	179	21.4	175	20.9
45+ years	68	8.1	73	8.8
Among female clients: Pregnancy and breastfeeding status[b]				
Pregnant	16	4.3	17	4.5
Breastfeeding	37	10.0	39	10.3
Not pregnant or breastfeeding	317	85.7	314	83.1
Missing (not indicated on form)	7		8	
Among male clients: Circumcision status[c]				
Circumcised	193	42.9	151	32.9
Missing (not indicated on form)	8		7	

Current STI or STI in the past 6 months	521	62.4	523	62.5
Partner with unsuppressed HIV viral load	390	46.7	389	46.5
Buys or sells sex	256	30.7	260	31.0
Among adolescent girls and young women (age 15-24): Partner 5+ years older[d]	41	33.9	25	20.3
Number of PrEP indications[e]				
0	38	4.6	38	4.6
1	414	49.6	411	49.1
2	355	42.5	362	43.2
3	28	3.4	26	3.1
4	0	0.0	0	0.0
Received PrEP readiness education	819	98.1	822	98.2
Acute HIV infection assessment				
Acute infection not suspected	188	23.2	163	20.2
No assessment done	624	76.8	646	79.8
Missing (not indicated on form)	23		28	
Kidney risk assessment[f]				
Screened; determination of high risk	0	0.0	0	0.0
Screened; determination of low risk	109	13.4	114	14.1
Not screened	702	86.6	694	85.9
Missing (not indicated on form)	24		29	
Hepatitis B testing				
Reactive result	1	0.1	1	0.1
Non-reactive result	833	99.8	836	99.9
No test administered	1	0.1	0	0.0

PrEP: Pre-exposure prophylaxis; STI: Sexually transmitted infection.

[a] ePrEP participants received enhanced STI services (i.e., baseline and quarterly etiologic STI testing and assisted partner notification) in addition to the standard-of-care services. They also completed bio-behavioral surveys and received financial incentives.

[b] Denominator for percentages: female clients.

[c] Denominator for percentages: male clients.

[d] Denominator for percentages: adolescent girls and young women (female clients ages 15-24).

[e] Among: Buys or sells sex, current STI or STI in the past 6 months, partner with unsuppressed HIV viral load, or adolescent girl or young woman with partner 5+ years older.

[f] Malawi PrEP guidelines call for baseline creatine clearance testing prior to PrEP initiation for clients age > 50, hypertension, diabetes mellitus, body mass index < 18.5, other nephrotoxic medication, or any signs or symptoms suggestive of renal impairment.

Data are from routine PrEP client cards for 835 clients who newly initiated PrEP at an STI clinic in Lilongwe, Malawi in March-December 2022.

* The unstabilized weight (u) for each standard-of-care recipient (i) was computed as:

$$u_i = \frac{1}{P(A = 1 | b_i, c_i, d_i, g_i, h_i, j_i)}$$

The mean of the unstabilized weights was 1.26 (min = 1.01, max = 2.60).
