# Supplementary material for: Poor Oral HIV Pre-Exposure Prophylaxis (PrEP) Persistence in an Integrated PrEP/STI Program in Malawi
Source: AIDS Behav. 2025 Nov 29;30(5):1327–37. doi: 10.1007/s10461-025-04937-y (PMC13167809; doi:10.1007/s10461-025-04937-y)
Supplement: Supplementary file 3 — Supplementary Material 3 [file 10461_2025_4937_MOESM3_ESM.pdf]

Mulholland GE, Matoga M, Chen JS, Mathiya E, Bell GJ, Ndalama B, Munthali T, Nyirenda N, Bonongwe N, Pedersen C, Jere E, Hosseinipour MC, Mphande Z, Hoffman IF, Rutstein SE. Poor oral HIV pre-exposure prophylaxis persistence in an integrated PrEP/STI program in Malawi. AIDS and Behavior.

Corresponding author: Grace E. Mulholland (gem@unc.edu); Department of Epidemiology, University of North Carolina at Chapel Hill, Chapel Hill, North Carolina, United States

Online Resource 3. Sex-stratified PrEP persistence estimates by PrEP indication

The table below presents PrEP persistence estimates according to PrEP indication, disaggregated by sex. Due to sparse data, it was not possible to estimate PRs specifically among female clients with PrEP indications of a partner with an unsuppressed HIV viral load at 6 months and buys or sells sex at 3 and 6 months.

Table. PrEP persistence by baseline PrEP indication and sex. The percentage of male and female clients persisting on PrEP under Malawi's standard-of-care PrEP services was estimated using routine records data from 835 clients newly initiating PrEP at an integrated PrEP program in March-December 2022. Persistence was defined as less than 7 days not covered by PrEP since a prior PrEP visit. Data are from the Enhanced PrEP/STI Study in Lilongwe, Malawi (2022-2023).

	Female clients			Male clients		
	**Percentage of clients persistent (95% CI)[a] at:**			**Percentage of clients persistent (95% CI) at:**		
Baseline PrEP indication	1 month	3 months	6 months	1 month	3 months	6 months
STI at PrEP initiation visit or in the prior 6 months						
Yes	11.2 (7.1, 17.7)	5.1 (2.6, 10.0)	2.4 (0.9, 6.5)	10.0 (6.8, 14.7)	4.5 (2.5, 8.0)	2.4 (1.1, 5.4)
No	28.6 (22.7, 36.1)	10.1 (6.5, 15.6)	5.0 (2.7, 9.5)	26.1 (19.6, 34.9)	14.6 (9.6, 22.1)	7.0 (3.7, 13.1)
Partner with unsuppressed HIV viral load						
Yes	27.2 (21.8, 34.0)	11.5 (7.9, 16.8)	6.3 (3.7, 10.6)	26.7 (20.6, 34.7)	13.9 (9.4, 20.6)	7.1 (4.0, 12.6)
No	8.6 (4.8, 15.5)	1.6 (0.4, 6.7)	0[b]	7.8 (4.8, 12.5)	3.7 (1.9, 7.4)	1.8 (0.7, 4.8)

Buys or sells sex						
Yes	1.8 (0.3, 12.5)	0	0	6.5 (3.4, 12.3)	2.0 (0.6, 6.2)	1.4 (0.3, 5.6)
No	22.9 (18.5, 28.4)	8.9 (6.1, 12.9)	4.4 (2.6, 7.5)	20.9 (16.3, 27.0)	11.6 (8.1, 16.6)	5.5 (3.2, 9.4)
Among adolescent girls or young women (ages 15-24): Partner 5+ years older						
Yes	12.4 (4.6, 33.3)	2.5 (0.4, 17.7)	2.5 (0.4, 17.7)	N/A	N/A	N/A
No	13.6 (7.5, 24.6)	5.8 (2.2, 15.4)	1.2 (0.2, 8.7)	N/A	N/A	N/A
	**Persistence probability ratio (95% CI) at:**			**Persistence probability ratio (95% CI) at:**		
	1 month	3 months	6 months	1 month	3 months	6 months
STI at PrEP initiation visit or in the prior 6 months						
Yes	0.39 (0.25, 0.60)	0.50 (0.26, 0.99)	0.49 (0.19, 1.27)	0.38 (0.26, 0.55)	0.31 (0.18, 0.53)	0.35 (0.16, 0.76)
No	1.00	1.00	1.00	1.00	1.00	1.00
Partner with unsuppressed HIV viral load‡						
Yes	3.17 (1.88, 5.34)	7.03 (2.12, 23.34)	–	3.44 (2.26, 5.24)	3.77 (2.05, 6.95)	4.02 (1.66, 9.73)
No	1.00	1.00	–	1.00	1.00	1.00
Buys or sells sex						
Yes	0.08 (0.02, 0.39)	–	–	0.31 (0.18, 0.53)	0.17 (0.07, 0.43)	0.25 (0.08, 0.80)
No	1.00	–	–	1.00	1.00	1.00
Among adolescent girls or young women (ages 15-24): Partner 5+ years older						
Yes	0.91 (0.35, 2.38)	0.43 (0.07, 2.77)	2.02 (0.20, 20.84)	N/A	N/A	N/A
No	1.00	1.00	1.00	N/A	N/A	N/A

CI: Confidence interval; PrEP: Pre-exposure prophylaxis; STI: Sexually transmitted infection.

[a] Data were reweighted to reflect the baseline distribution of age, sex, and PrEP indication among all 835 PrEP initiators, and robust standard errors were used in computing 95% confidence intervals (presented parenthetically).

[b] Values of 0 for percentage persistent indicate that no clients in the stratum persisted on PrEP to the indicated time point.

[c] Dashes (–) indicate comparisons where, within one or both levels of the characteristic, 0 clients persisted on PrEP.

Data are from routine PrEP client cards for 835 clients who newly initiated PrEP at an STI clinic in Lilongwe, Malawi in March-December 2022.
