# Supplementary material for: Poor Oral HIV Pre-Exposure Prophylaxis (PrEP) Persistence in an Integrated PrEP/STI Program in Malawi
Source: AIDS Behav. 2025 Nov 29;30(5):1327–37. doi: 10.1007/s10461-025-04937-y (PMC13167809; doi:10.1007/s10461-025-04937-y)
Supplement: Supplementary file 4 — Supplementary Material 4 [file 10461_2025_4937_MOESM4_ESM.pdf]

Mulholland GE, Matoga M, Chen JS, Mathiya E, Bell GJ, Ndalama B, Munthali T, Nyirenda N, Bonongwe N, Pedersen C, Jere E, Hosseinipour MC, Mphande Z, Hoffman IF, Rutstein SE. Poor oral HIV pre-exposure prophylaxis persistence in an integrated PrEP/STI program in Malawi. AIDS and Behavior.

Corresponding author: Grace E. Mulholland (gem@unc.edu); Department of Epidemiology, University of North Carolina at Chapel Hill, Chapel Hill, North Carolina, United States

Online Resource 4. Sensitivity of persistence estimates to availability of missed PrEP doses

The first row in the figure below presents the persistence estimates as calculated in the main text, where a client's expected PrEP supply assumes that missed PrEP doses reported at a visit remained available to the client. The second row presents the persistence estimates as computed under the assumption that missed PrEP doses reported at a visit were not available to the client.

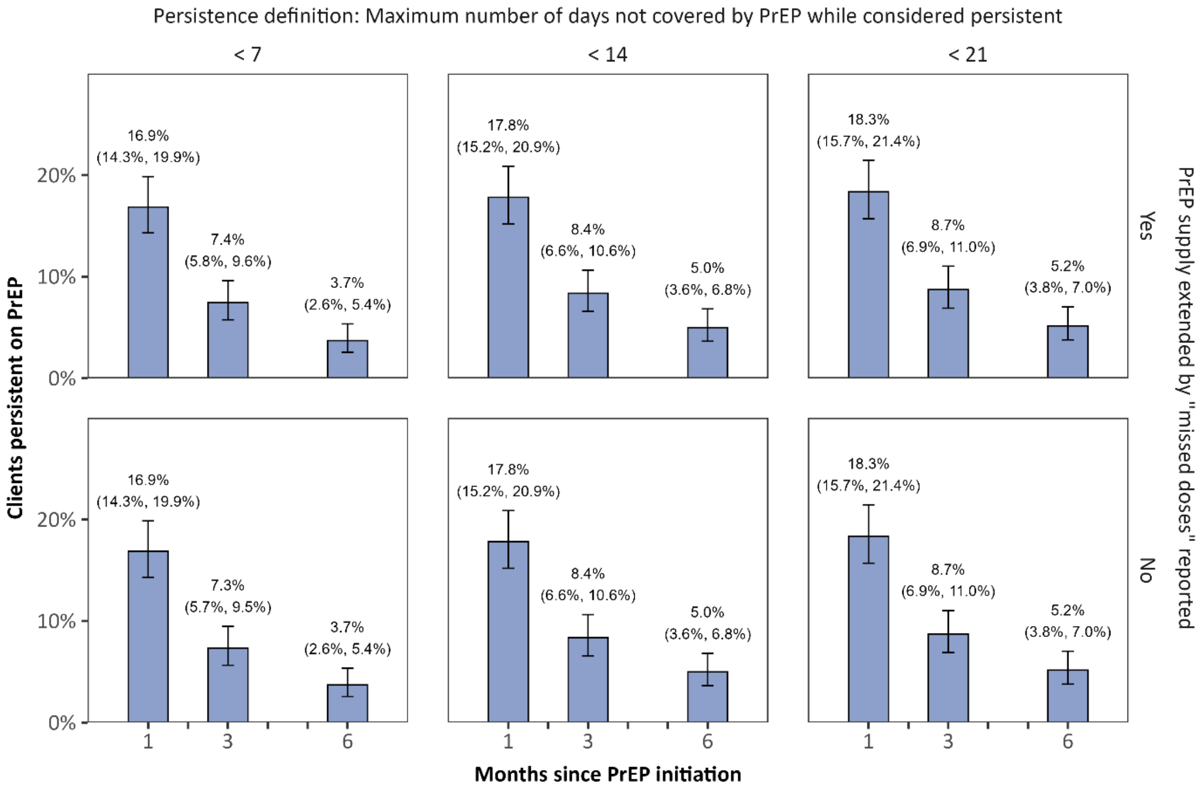

Fig. PrEP persistence under 3 persistence definitions and by inclusion vs. exclusion of missed PrEP doses when computing PrEP supply. Bars show the percentage of clients estimated to

persist on PrEP under Malawi’s standard-of-care PrEP services when persistence is defined as less than 7, 14 or 21 days not covered by PrEP since a prior PrEP visit. Data were reweighted to reflect the baseline distribution of age, sex, and PrEP indication among all 835 PrEP initiators, and robust standard errors were used in computing 95% confidence intervals (indicated by error bars).
